# Ag Nanoparticles Drop-Casting Modification of Screen-Printed Electrodes for the Simultaneous Voltammetric Determination of Cu(II) and Pb(II)

**DOI:** 10.3390/s17061458

**Published:** 2017-06-21

**Authors:** Clara Pérez-Ràfols, Julio Bastos-Arrieta, Núria Serrano, José Manuel Díaz-Cruz, Cristina Ariño, Joan de Pablo, Miquel Esteban

**Affiliations:** 1 Departament d’Enginyeria Química i Química Analítica, Facultat de Química, Universitat de Barcelona, Martí i Franquès 1-11, 08028 Barcelona, Spain; claraperezrafols@ub.edu (C.P.-R.); nuria.serrano@ub.edu (N.S.); cristina.arino@ub.edu (C.A.); miquelestebanc@ub.edu (M.E.); 2 Departament d’Enginyeria Química, Universitat Politècnica de Catalunya (UPC), Campus Diagonal Besòs, Edificio I (EEBE), Carrer Eduard Maristany 10-14, 08019 Barcelona, Spain; julio.bastos@upc.edu (J.B.-A.); joan.de.pablo@upc.edu (J.d.P.); 3 Barcelona Research Center for Multiscale Science and Engineering, 08019 Barcelona, Spain; 4 Fundació CTM Centre Tecnològic, Plaça de la Ciència 2, 08240 Manresa, Spain

**Keywords:** silver nanoparticles, screen-printed electrodes, anodic stripping voltammetry, metal ions, nanoparticle drop-casting

## Abstract

A new silver nanoparticle modified screen-printed electrode was developed and applied to the simultaneous determination of Pb(II) and Cu(II). Two different types of silver nanoparticles with different shapes and sizes, Ag nanoseeds and Ag nanoprisms, were microscopically characterized and three different carbon substrates, graphite, graphene and carbon nanofibers, were tested. The best analytical performance was achieved for the combination of Ag nanoseeds with a carbon nanofiber modified screen-printed electrode. The resulting sensor allowed the simultaneous determination of Pb(II) and Cu(II) at trace levels and its applicability to natural samples was successfully tested with a groundwater certified reference material, presenting high reproducibility and trueness.

## 1. Introduction

Voltammetric stripping techniques have been widely applied to trace metal ions determination because they present excellent detection limits, great sensitivity to the presence of different metals and capacity to multimetal determination with a relatively low cost [1]. For several decades, these techniques mainly used the hanging mercury drop electrode (HMDE) as a working electrode. HMDE presents several advantages such as new drops being formed continuously, which avoids problems related to contamination, its great reproducibility or its extensive cathodic potential range (from +0.4 V to −2.5 V according to supporting electrolyte) among others [2]. However, potential toxicity risks associated to mercury use and disposal, together with the difficulty to adapt HMDE to sensor devices and flow systems, have led to the introduction of new working electrode materials throughout the years.

Bismuth film electrodes (BiFE) were proposed in 2000 as an alternative to HMDE since they offered properties close to those of mercury with the advantages of being more environmentally friendly and easily adaptable to sensor devices and flow systems [3]. BiFE, though, presents a narrower anodic potential range since the stripping peak of bismuth appears at −0.3 V. This fact prevents the use of BiFE prepared via ex situ plating or preplated method to the determination of more easily oxidizable metals like Cu(II), whereas the in situ BiFE approach, where Bi(III) ions are electrochemically deposited on the electrode surface during the analysis, cannot be used to the determination of copper either because Bi(III) and Cu(II) ions compete for the surface sites [4,5]. 

The anodic potential range was slightly extended with the introduction of antimony film electrodes (SbFE) in 2007 since the stripping peak of antimony does not appear until −0.1 V [6]. This potential range, though, is still not wide enough to allow the determination of copper with an ex situ SbFE approach. Better results were obtained with an SbFE prepared via in situ, where Cu(II) was successfully determined [7]. However, the use of in situ SbFEs implies the addition of Sb(III) ions to the sample solution, causing a slight overlapping between both the Cu(II) and the Sb(III) peaks, which is not an ideal situation. 

Other types of working electrodes like solid electrodes or chemically modified electrodes (CME) have been successfully applied to the determination of Cu(II). These types of electrodes usually present a much wider anodic potential range than bismuth and antimony based electrodes. Nevertheless, CMEs usually present longer manufacturing times, whereas, when working with solid electrodes, it is difficult to control their surface in a reproducible manner and the peaks appear distorted and with excessive noise [8]. It should be pointed out that the use of under potential deposition (UPD) combined with stripping techniques for analytical applications can overcome some of the major disadvantages related to solid electrodes [9].

Another field that brings more possibilities to the development of improved electrochemical sensors is the use of nanomaterials. At this small scale, materials present some unique optical, mechanical, electrical and chemical properties and, compared to electrodes based on bulk materials, nanomaterials present some enhanced properties such as increased electrode surface area, increased mass-transport rate and faster electron transfer [10]. In this sense, nanostructured modified electrodes ranging from carbon-based nanomaterials to metallic nanoparticles have been studied for metal ion determination [10,11,12]. Concerning metallic nanoparticles, good results have been obtained using bismuth, antimony and gold among others [10,11]. Silver nanoparticles, on the other hand, are also being explored due to their good chemical and physical properties as well as their inexpensiveness. Some good results have already been reported for the determination of Sb(III) [13], Cr(VI) [14], Pb(II) and Cd(II) [15]. Furthermore, electrodes based on silver nanoparticles present a wider anodic potential range compared to SbFEs, which makes them good candidates for the determination of Cu(II). 

Silver nanoparticles electrodes can be based on different supports. In this sense, the appearance of the screen-printing technology has represented a huge progress thanks to the disposable character, good reproducibility and low-cost commercial availability of screen-printed devices. Moreover, the possibility of using a great diversity of compositions of printing inks, as well as the easy modification of their surface and the design versatility are important advantages of these devices [3,16]. These exciting benefits of screen-printed electrodes as supports over traditional based electrodes for metal ion determination have also been reported by Honeychurch et al. [17].

Inspired by both, the good properties of silver nanoparticles and the great features of screen-printed technology, in this work, we report for the first time the determination of Cu(II) with a silver-nanoparticle based- screen-printed electrode. In this reported electrode silver nanoparticles, Ag-nanoseeds and Ag-nanoprisms were simply prepared and easily incorporated to commercially available screen-printed electrodes. These silver-nanoparticles were microscopically and analytically studied. Furthermore, the determination of Pb(II) and its interaction with Cu(II) have also been considered. Additionally, the simultaneous determination of Pb(II) and Cu(II) in a certified groundwater sample was successfully achieved.

## 2. Materials and Methods 

### 2.1. Chemicals

Certified reference material, groundwater (BCR^®^-610), was provided by Sigma-Aldrich (St. Louis, MO, USA). All other reagents used were purchased from Panreac (Barcelona, Spain) and Merck (Darmstadt, Germany) analytical grade. Daily Cu(II) and Pb(II) standard solutions were prepared by appropriate dilution of stock solutions 10^−2^ mol·L^−1^, prepared from Cu(NO_3_)_2_·3H_2_O and Pb(NO_3_)_2_·4H_2_O, respectively, and complexometrically standardized [18]. Acetate buffer 0.1 mol·L^−1^ (pH 4.5) was used for pH control and ultrapure water (Milli-Q plus 185 system, Millipore, Billerica, MA, USA) was used in all experiments.

Silver nitrate, poly(sodium styrenesulphonate) (PSSS), trisodium citrate, sodium borohydride and ascorbic acid were used for the preparation of Ag-nanoseeds and Ag-nanoprims, all from Sigma-Aldrich (St. Louis, MO, USA). 

### 2.2. Apparatus

For stripping voltammetric measurements, an Autolab System PGSTAT12 (EcoChemie, Utrecht, the Netherlands), in its multichannel configuration, attached to a Metrohm 663 VA Stand (Metrohm, Herisau, Switzerland) was used. GPES Multichannel 4.7 software package (EcoChemie, Utrecht, The Netherlands) was employed for data acquisition.

Pt wire and Ag|AgCl|KCl (3 mol·L^−1^) (Metrohm, Herisau, Switzerland) were used as auxiliary and reference electrodes, respectively. The working electrode was either a carbon screen-printed electrode (SPCE), a carbon nanofiber modified screen-printed electrode (SPCNFE) or a graphene modified screen-printed electrode (SPGPHE) modified with silver nanoparticles and connected to the Autolab Systems by means of a flexible cable (ref. CAC, DropSens). SPCE, SPCNFE and SPGPHE were disk electrodes of 4 mm diameter purchased from Dropsens (Oviedo, Spain) (ref. 110, DS SPCE, ref. 110CNF, DS SPCE and ref. 110GPH, DS SPCE respectively).

A pH-meter Basic 20 (Crison Instruments, Barcelona, Spain) was used for pH measurements.

Surface morphology characterization was performed by a scanning electron microscope JSM 7100FE from JEOL (Tokyo, Japan).

Transmission Electron Microscopy (TEM) images were obtained using a JEM Jeol 2100 microscope (Peabody, MA, USA) at 200 kV, coupled with an Energy-Dispersive Spectrometer (Inca X-Sight from Oxford Instruments, Abingdon, UK).

### 2.3. Synthesis and Characterization of Ag Nanoseeds and Ag Nanoprisms

The preparation of different types of Ag nanoparticles was carried out by Seed Mediated Approach Methodology [19,20]. Seed nucleation is due to NaBH_4_ (as strong reducing agent) that leads to the formation of small spherical nanoparticles, with trisodium citrate and PSSS as stabilizers. For nanoprism formation, ascorbic acid acts as reducing agent (softer than NaBH_4_) with preferential axial growth in crystallization. This fact leads to the formation of the shape defined nanoprisms, with sodium tricitrate used as nanoprism stabilizer. Thus, Ag nanoseeds were produced by combining aqueous trisodium citrate (5 mL, 2.5 mmol·L^−1^), aqueous PSSS (0.25 mL, 500 mg·L^−1^) and aqueous NaBH_4_ (0.3 mL, 10 mmol·L^−1^, freshly prepared) followed by addition of aqueous AgNO_3_ (5 mL, 0.5 mmol·L^−1^) at a rate of 2 mL·min^−1^ while stirring continuously. Then, a specific Ag nanoprism sample was prepared by combining 5 mL distilled water, aqueous ascorbic acid (75 μL, 10 mmol·L^−1^) and 2.5 mL of the previously synthesized Ag nanoseeds, followed by addition of aqueous AgNO_3_ (3 mL, 0.5 mmol·L^−1^) at a rate of 1 mL·min^−1^. After synthesis, aqueous trisodium citrate (0.5 mL, 25 mmol·L^−1^) was added to stabilize the particles. Milli-Q Water was used throughout all solutions.

The size distribution of the prepared nanoparticles was obtained by direct observation of the TEM images and the construction of the corresponding size distribution histograms. These measurements were performed using the ImageJ Software (1.48 V), and the histograms obtained were adjusted to a three-parameter Gaussian curve (Equation (1)), where *x*_0_ is the mean diameter (to which most nanoparticles correspond), *b* is the standard deviation and *a* is a statistical parameter related to this fitting. Histograms were calculated using Excel 2010 (Microsoft, Redmond, WA, USA), and the equation was adjusted with SigmaPlot 11.0 (Systat Software, San José, CA, USA). For Ag nanoprisms, heights were used as reference for the diameter value:

(1) $$y=ae^{\left[-0.5{\left(\frac{x_{0}-x}{b}\right)}^{2}\right]}.$$

### 2.4. Preparation of Ag-Nanoparticles-SPCNFE

Ag-nanoparticles-SPCNFE was prepared by drop-casting 40 μL of silver nanoparticles solution onto the electrode surface and drying it for 30 min at 50 °C.

### 2.5. Voltammetric Measurements

For voltammetric stripping determinations of Pb(II) and Cu(II) using Ag-nanoparticles-SPCNFE, both metal ions were deposited at a deposition potential (E_d_) of −1.1 V, applied with stirring, during a deposition time (t_d_) of 120 s. Deposition was followed by a rest period (t_r_) of 5 s and then the potential was scanned from −1.1 to 0.15 V using pulse times of 50 ms, step potentials of 5 mV and pulse amplitudes of 50 mV.

Linear calibration plots for both separate and simultaneous determinations of Cu(II) and Pb(II) were performed by increasing metal ion concentrations in 0.1 mol·L^−1^ acetate buffer (pH 4.5).

The analysis of the certified groundwater sample was performed by the standard addition calibration method. First of all, a volume of the sample (BCR^®^-610) was adjusted to pH 4.5 with sodium acetate and the scan was recorded. Then, four aliquots of Cu(II) and Pb(II) standard solutions were added and the respective curves were recorded.

## 3. Results and Discussion

### 3.1. Electron Microscopy Characterization of Ag-Nanoparticles-SPCNFE

**Figure 1:**
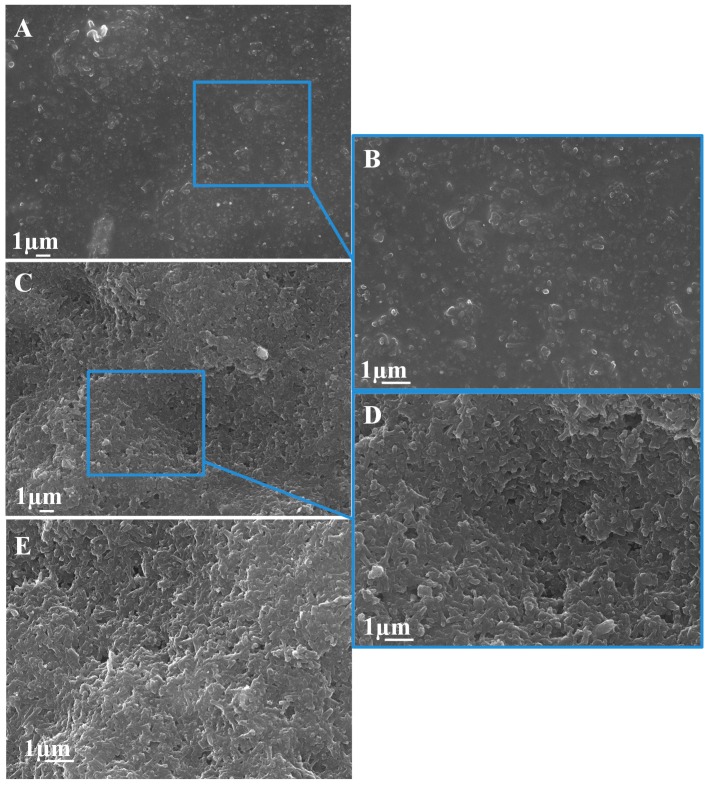
SEM micrographs of Ag-nanoseeds-SPCNFE (**A**,**B**), Ag-nanoprisms-SPCNFE (**C**,**D**) and bare SPCNFE (**E**). Magnification of E = A = C <<< B = D.

The incorporation of nanoparticles (NPs) in sensing systems derives to the enhancement and customization of the electrochemical features (e.g., oxidation or reduction current onto a transducing platform), which offers novel option for electroanalytical purposes in environmental and biological fields [21]. Consequently, the incorporation of Ag-NPs to commercially available screen-printed electrodes implies the increase of electrocatalytically active zones on the structure of the composite material. SEM images of the surface of SPCNFE modified with Ag-nanoseeds (Figure 1A,B) and Ag-nanoprisms (Figure 1C,D) show, in comparison to the bare SPCNFE (Figure 1E), the dispersion of these NPs all over the electrode surface (white dots). This can explain the capability to improve the electrochemical performance in comparison with the non–modified SPCNFE, as previously observed in other electrochemical systems modified with nanomaterials [22].

**Figure 2:**
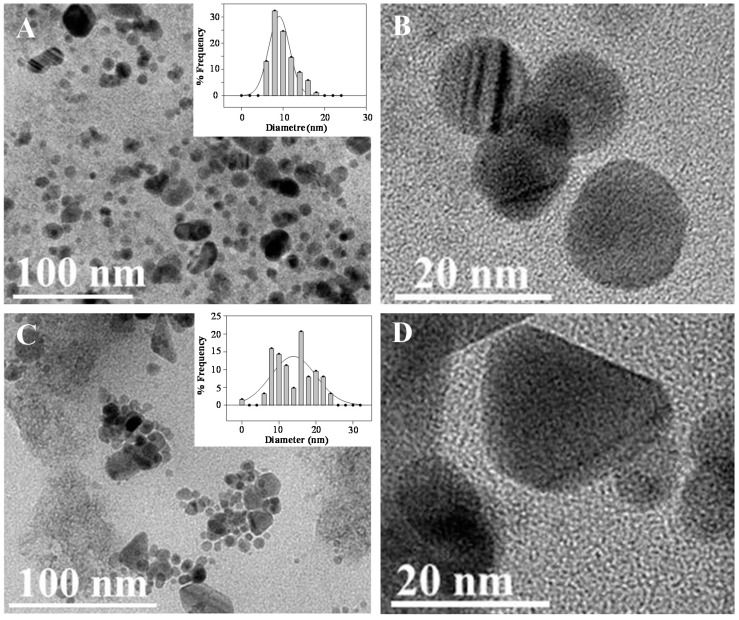
TEM micrographs of 9.1 ± 0.4 nm spherical Ag-nanoseeds (**A**,**B**) and triangular shaped 14.0 ± 0.9 nm Ag-nanoprisms (**C**,**D**) with the corresponding size distribution histograms (insets in **A** and **C**). Magnification of A = C <<< B = D.

Seed mediated approach synthesis of nanostructures is a multistep methodology [23] based on the first fast crystallization of nanoseeds, which can be used for further nucleation and customized growth of shape defined nanoprisms of larger size [24]. TEM characterization of spherical Ag-nanoseeds (Figure 2A,B) and shaped defined Ag-nanoprisms (Figure 2C,D) provides valuable morphological information of the materials incorporated to SPCNFE matrix.

### 3.2. Enhanced Modification of SPE with Ag-Nanoparticles

The attachment of the silver nanoparticles to the screen-printed electrodes surface was firstly optimized using Ag-nanoseeds as a model of silver nanoparticles. Different drop volumes and drying times were tested and the best results were obtained by depositing a single drop of 40 μL of the Ag-nanoseeds solution to the electrode surface and drying it in the oven at 50 °C.

**Figure 3:**
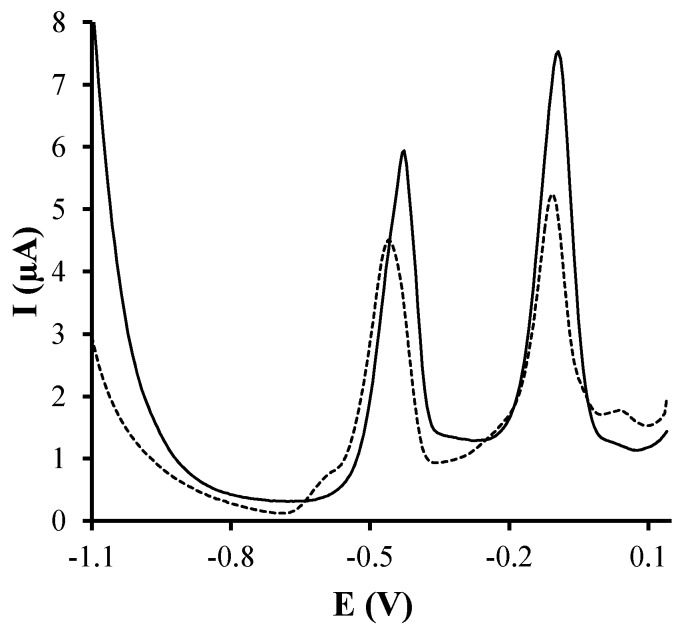
Stripping voltammetric measurements of a solution containing 100 μg·L^−1^ of Cu(II) and Pb(II) at pH 4.5 using an E_d_ of −1.1 V during a t_d_ of 120 s on Ag-nanoseeds-SPCE (solid line) and SPCE (dashed line).

In order to see the enhancement of the electrochemical response provided by the silver nanoparticles screen-printed electrode, firstly, the measurements of a solution containing 100 μg·L^−1^ of Cu(II) and Pb(II) were carried out on both SPCE and Ag-nanoseeds-SPCE. As it can be seen in Figure 3, the attachment of the silver nanoparticles on the screen-printed surface considerably increases the voltammetric response for both considered metal ions, suggesting that a sensor based on the modification with Ag-nanoparticles could be a better alternative for the determination of Cu(II) and Pb(II).

Taking into account that it has been reported that the use of carbon based nanomaterials supports improves the analytical performance of sensors [25], three different carbon-based screen-printed substrates, graphite, graphene and carbon nanofibers, were compared prioritizing the repeatability and reproducibility of the resulting sensor. In this sense, stripping measurements of a solution containing 100 μg·L^−1^ of Cu(II) and Pb(II) in acetate buffer pH 4.5 were carried out. The repeatability was calculated from 10 repetitive measurements using the same Ag-nanoseeds-SPE, whereas the reproducibility was measured from three different Ag-nanoseeds-SPE units within a series of 10 repetitive measurements. Ag-nanoseeds-SPCE was the least repetitive sensor, with relative standard deviations (RSD) yielding 18.1% for Cu(II) and 20.1% for Pb(II). Better repeatability results were obtained for Ag-nanoseeds-SPCNFE (3.6% for Cu(II) and 5.5% for Pb(II)) and Ag-nanoseeds-SPGPHE (3.2% for Cu(II) and 3.1% for Pb(II)). This fact could be attributed to the enlarged surface area and rugosity provided by the nanostructured carbon materials, which help retaining the Ag-nanoseeds onto the electrode surface. 

Given the low repeatability observed for Ag-nanoseeds-SPCE, this substrate was discarded and the reproducibility was studied for both Ag-nanoseeds-SPCNFE and Ag-nanoseeds-SPGPHE. In this case, Ag-nanoseeds-SPCNFE provided better results (5.2% for Cu(II) and 8.1% for Pb(II)) compared to Ag-nanoseeds-SPGPHE (19.3% for Cu(II) and 15.2% for Pb(II)). This poor reproducibility values obtained for Ag-nanoseeds-SPGPHE could be attributed to the low homogeneity between the different units commercially purchased. This fact was previously observed for the preparation of antimony film coated on SPGPHE [26].

In view of these results, SPCNFE was selected as the optimal carbon substrate and its repeatability and reproducibility were also studied for Ag-nanoprisms, yielding 3.6% and 6.3% for Cu(II) and 5.1% and 9.3% for Pb(II), respectively. As it can be seen, similar repeatability and reproducibility were obtained for both Ag-nanoseeds and Ag-nanoprisms. Furthermore, these reproducibility values are also in agreement with reproducibility values reported with silver nanoparticles modified electrodes for Sb(III) [13], Pb(II), Cd(II) [15] and Cr(IV) [14].

### 3.3. Electrochemical Parameters and Calibration Data

Once the preparation of the silver nanoparticle modified electrodes was optimized, different electrochemical parameters were optimized for the simultaneous determination of Pb(II) and Cu(II). In this sense, stripping measurements of a solution containing 100 μg·L^−1^ of Cu(II) and Pb(II) in acetate buffer pH 4.5 were carried out at several deposition potentials (E_d_) ranging from −0.6 to −1.2 V and using a deposition time (t_d_) of 120 s as a compromise between peak area and analysis time. When applying E_d_ between −0.6 V and −0.9 V, two overlapped peaks were observed in the copper oxidation region. The reasons for such behavior are not clear but could be related to the stabilization of Cu(I) onto the electrode surface. Anyway, using more negative E_d_, a single peak clearly corresponding to the Cu(II) oxidation was observed. This peak increased when the E_d_ was shifted from −0.95 to −1.1 V and decreased at more negative potentials. Therefore, an E_d_ of −1.1 V was selected as the optimal for the determination of Cu(II) and Pb(II).

The anodic range of the Ag-nanoparticles-SPCNFE was also studied. It was observed that the stripping peak associated to Ag/Ag(I) did not appear until 0.2 V, which perfectly allows the determination of Cu(II) which appears at ca. −0.11 V. It should be pointed out that this anodic range is wider than that provided by a silver screen-printed electrode where the stripping peak associated to the oxidation of Ag appears at ca. −0.03 V. This fact is in agreement with the behavior showed by a rotating silver electrode [25], where Cu(II) cannot either be determined and could be attributed to nanomaterials having different redox potentials than bulk materials [27].

**Figure 4:**
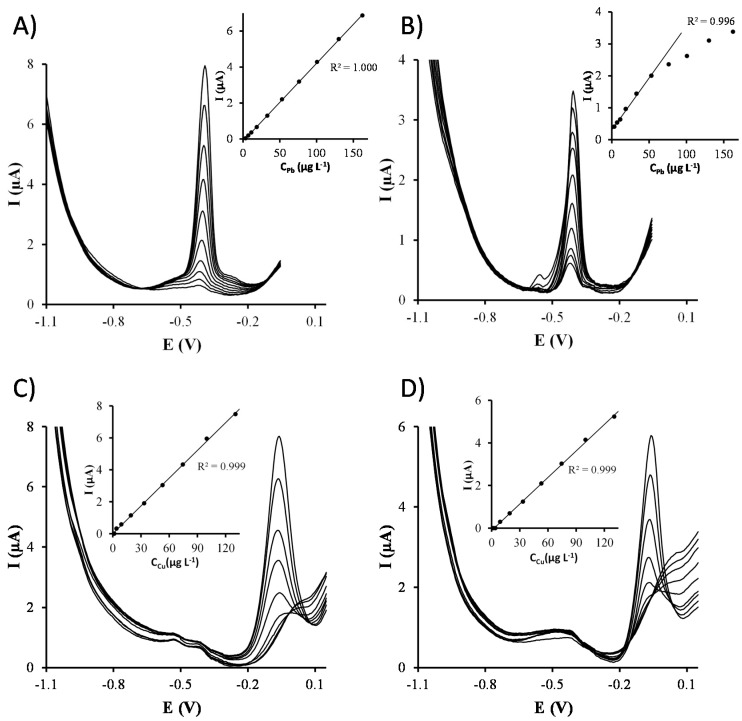
Stripping voltammetric measurements and calibration curves (insets) obtained for the individual calibration of Pb(II) (**A**,**B**) and Cu(II) (**C**,**D**) in acetate buffer pH 4.5 using an Ag-nanoseeds-SPCNFE (**A**,**C**) and an Ag-nanoprisms-SPCNFE (**B**,**D**) at an E_d_ of −1.1 V and a t_d_ of 120 s.

Once the electrochemical parameters were optimized, separate calibrations of Cu(II) and Pb(II) were carried out on both Ag-nanoseeds-SPCNFE and Ag-nanoprisms-SPCNFE sensors (Figure 4). Table 1 reports the sensitivities, correlations, linear ranges and limits of detections (LOD) obtained. Sensitivities were calculated as the slope of the calibration curve, whereas LODs and LOQs were calculated as three and ten times, respectively, the standard deviation of the intercept over the slope. As it can be observed, Ag-nanoprisms-SPCNFE provides lower sensitivities compared to Ag-nanoseeds-SPCNFE, which also presents slightly better LODs. This fact could be attributed to the spherical shape of Ag-nanoseeds, which provides a larger surface area, as well as to the wetting behavior of the nanoparticle containing solutions that leads to a more homogeneously distributed layer. Nevertheless, in both cases, the obtained LODs are similar or even better than those provided in the literature using CMEs [28,29], in situ SbFE [7,30,31] or silver nanoparticles based electrodes [32] for Cu(II) and CMEs [28,33,34], SbFEs [6], BiFEs [3] or silver nanoparticles based electrodes [15,32] for Pb(II). It should also be pointed out that, although the linear range for Cu(II) is similar for both Ag-nanoseeds-SPCNFE and Ag-nanoprisms-SPCNFE, in the case of Pb(II), the electrode surface is easily saturated when the Ag-nanoprisms-SPCNFE is used, which results in a significantly narrower linear range.

**Figure 5:**
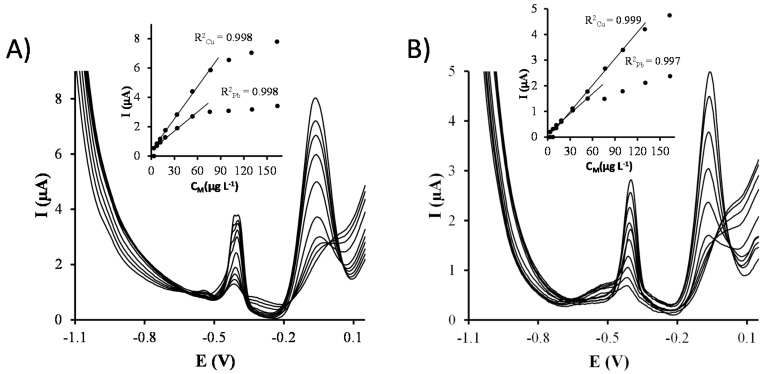
Stripping voltammetric measurements and calibration curves (insets) obtained for the simultaneous calibration of Pb(II) and Cu(II) in acetate buffer pH 4.5 using an Ag-nanoseeds-SPCNFE (**A**) and an Ag-nanoprisms-SPCNFE (**B**) at an E_d_ of −1.1 V and a t_d_ of 120 s.

In order to study the interaction between Cu(II) and Pb(II) ions, simultaneous calibrations of both target metal ions were also carried out on Ag-nanoseeds-SPCNFE and Ag-nanoprisms-SPCNFE (Figure 5 and Table 1). It can be observed that both metals interact with each other, resulting in changes of the sensitivities as compared to separate calibrations. The most notorious thing, though, is the shortening of the linearity range probably caused by the competition of Cu(II) and Pb(II) for the active sites of the electrode surface. 

Although both Ag-nanoseeds-SPCNFE and Ag-nanoprisms-SPCNFE present a similar analytical performance regarding repeatability, reproducibility, linear range and LODs, the higher sensitivities achieved by Ag-nanoseeds-SPCNFE together with the fact that Ag-nanoseeds require fewer steps to be synthesized than Ag-nanoprisms, Ag-nanoseeds-SPCNFE was selected to test its applicability in natural samples.

### 3.4. Application to the Analysis of a Groundwater Reference Material

**Figure 6:**
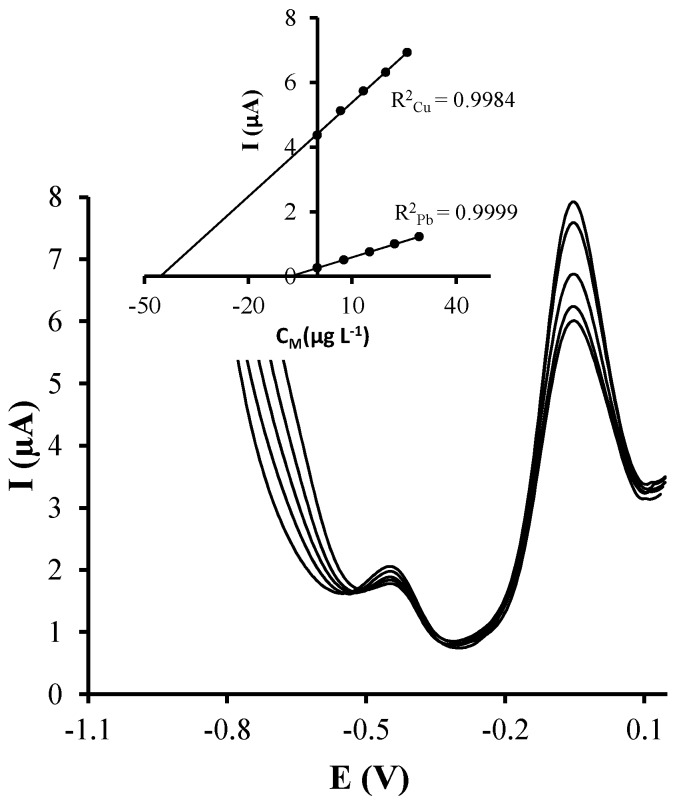
Stripping voltammetric measurements in groundwater samples on Ag-nanoseeds-SPCNFE at pH 4.5 using an E_d_ of −1.1 V during a t_d_ of 120 s. Inset: Pb(II) and Cu(II) standard calibration plots.

In view of the above discussed results, Ag-nanoseeds-SPCNFE was considered for the determination of Cu(II) and Pb(II) in natural samples. Thus, the applicability of this sensor was tested by simultaneously determining Pb(II) and Cu(II) in a groundwater certified reference material (BCR^®^-610). Figure 6 shows representative voltammograms for the simultaneous analysis of Cu(II) and Pb(II) in the groundwater reference material. Pb(II) and Cu(II) concentrations were determined using the standard addition method, and, as it can be observed, well defined peaks were obtained for both target ions like in the calibration samples. 

Calibration curves for both Pb(II) and Cu(II) are shown in the inset of Figure 6. Good correlations were obtained in both cases. Three replicates of the simultaneous voltammetric determination of Cu(II) and Pb(II) in the groundwater certified material using Ag-nanoseeds-SPCNFE were carried out. The obtained concentration data is reported in Table 2. As it can be observed, good concordance between the replicates, inferred by the relative standard deviation (RSD), and with the certified metal ion concentration values were achieved. It should be mentioned that this groundwater material also contains other metal ions including cadmium, arsenic, aluminium and nickel, which do not seem to interfere in the voltammetric determination of Cu(II) and Pb(II) using Ag-nanoseeds-SPCNFE.

The achieved results confirm the applicability of the developed Ag-nanoseeds-SPCNFE for the simultaneous determination of Cu(II) and Pb(II) in natural samples at μg·L^−1^ levels. Therefore, Ag-nanoseeds-SPCNFE could be an interesting and valuable alternative to more conventional electrodes for the determination of metal ions, particularly in the case of Cu(II) where antimony and bismuth electrodes present some problems.

## 4. Conclusions

In this work, two different types of silver nanoparticles, Ag-nanoseeds and Ag-nanoprisms, were microscopically characterized and considered for the modification of carbon-based screen-printed electrodes and their application to the determination of Cu(II) and Pb(II). It should be pointed out that, in contrast to more sophisticated modification strategies, the incorporation of the silver nanoparticles to the electrode surface is based on a fast and easy procedure where silver nanoparticles solution is directly drop-casted.

The resulting sensors were microscopically and analytically characterized and compared to each other. Three different carbon substrates were tested and SPCNFE was selected because it provided better repeatability and reproducibility. Concerning the type of silver nanoparticle, both Ag-nanoseeds-SPCNFE and Ag-nanoprisms-SPCNFE provided similar repeatability, reproducibility, linear range and LODs. However, Ag-nanoseeds-SPCNFE showed higher sensitivities and their synthesis is easier.

Furthermore, Ag-nanoseeds-SPCNFE was successfully applied to the simultaneous determination of Cu(II) and Pb(II) in a groundwater certified reference material, providing good reproducibility and trueness inferred by the RSD (2.94% for Pb(II) and 1.2% for Cu(II)) and the relative error (0.64% for Pb(II) and 0.2% for Cu(II)), respectively. Therefore, these good results confirm the applicability of Ag-nanoseeds-SPCNFE for the simultaneous determination of Cu(II) and Pb(II) in natural samples.

## Figures and Tables

**Table 1:** Calibration data for both separate and simultaneous determination of Pb(II) and Cu(II) on Ag-nanoseeds-SPCNFE and Ag-nanoprisms-SPCNFE at E_d_ of −1.1 V, t_d_ of 120 s and pH 4.5. The standard deviations are denoted by parenthesis.

	Separate		Simultaneous	
	Pb(II)	Cu(II)	Pb(II)	Cu(II)
**Ag-nanoseeds-SPCNFE**				
Sensitivity (nA·μg^−1^·L)	43.3 (0.2)	57.5 (0.7)	43 (1)	73 (2)
R^2^	1.000	0.999	0.998	0.998
Linear range ^1^ (μg·L^−1^)	3.2–162.5	7.6–130.7	6.6–53.5	10.0–77.0
LOD (μg·L^−1^)	0.96	2.29	1.98	2.99
**Ag-nanoprisms-SPCNFE**				
Sensitivity (nA·μg^−1^·L)	32.3 (0.9)	41.4 (0.6)	25.9 (0.7)	34.4 (0.5)
R^2^	0.996	0.999	0.997	0.999
Linear range ^1^ (μg·L^−1^)	7.3–53.1	9.9–130.7	7.8–53.5	8.3–100.7
LOD (μg·L^−1^)	2.20	2.98	2.35	2.49

^1^ The lowest value of the linear range was considered from LOQ.

**Table 2:** Total concentrations of Pb(II) and Cu(II) determined in certified groundwater (BCR^®^-610) by stripping voltammetry on Ag-nanoseeds-SPCNFE by the standard addition calibration method applying an E_d_ of −1.1 V and t_d_ of 120 s at pH 4.5.

	Lead(II)			Copper(II)		
	c (μg·L^−1^)	RSD (%)	Relative Error (%)	c (μg·L^−1^)	RSD (%)	Relative Error (%)
**Ag-nanoseeds-SPCNFE**	7.83	2.94	0.64	45.6	1.2	0.2
**Certified metal value**	7.78	1.67	—	45.7	3.3	—
